# The Usefulness of Electronic Health Records From Preventive Youth Healthcare in the Recognition of Child Mental Health Problems

**DOI:** 10.3389/fpubh.2021.658240

**Published:** 2021-05-31

**Authors:** Nynke R. Koning, Frederike L. Büchner, Anouk W. van den Berg, S. Y. Angelique Choi, Nathalie A. Leeuwenburgh, Irma J. M. Paijmans, D. J. Annemarie van Dijk-van Dijk, Mattijs E. Numans, Mathilde R. Crone

**Affiliations:** ^1^Department of Public Health and Primary Care, Leiden University Medical Centre, Leiden, Netherlands; ^2^GGD Hollands Midden, Leiden, Netherlands

**Keywords:** mental health, pediatrics, public health, primary care, early identification

## Abstract

**Background and Objectives:** Early identification of child mental health problems (MHPs) is important to provide adequate, timely treatment. Dutch preventive youth healthcare monitors all aspects of a child's healthy development. We explored the usefulness of their electronic health records (EHRs) in scientific research and aimed to develop prediction models for child MHPs.

**Methods:** Population-based cohort study with anonymously extracted electronic healthcare data from preventive youth healthcare centers in the Leiden area, the Netherlands, from the period 2005–2015. Data was analyzed with respect to its continuity, percentage of cases and completeness. Logistic regression analyses were conducted to develop prediction models for the risk of a first recorded concern for MHPs in the next scheduled visit at age 3/4, 5/6, 10/11, and 13/14 years.

**Results:** We included 26,492 children. The continuity of the data was low and the number of concerns for MHPs varied greatly. A large number of determinants had missing data for over 80% of the children. The discriminatory performance of the prediction models were poor.

**Conclusions:** This is the first study exploring the usefulness of EHRs from Dutch preventive youth healthcare in research, especially in predicting child MHPs. We found the usefulness of the data to be limited and the performance of the developed prediction models was poor. When data quality can be improved, e.g., by facilitating accurate recording, or by data enrichment from other available sources, the analysis of EHRs might be helpful for better identification of child MHPs.

## Introduction

Despite having different healthcare systems, most high income countries provide some form of preventive child care that aims to monitor a child's healthy development during the first years of life (1–3). In the Netherlands, preventive well-child care is separated from curative care. Nurses and community pediatricians [preventive youth healthcare professionals (PYHPs)] provide free of charge preventive healthcare for all children aged 0 to 19 years during periodic health check-ups (4). The goal of these check-ups is to prevent disease, promote health and allow early identification of health risks, disease, and developmental problems (4). Over 80–90% of children are regularly seen in preventive youth healthcare (PYH) (5, 6). PYHPs work closely together with, amongst others, professionals in schools and in case of issues, PYHPs can provide additional advice or schedule extra visits, or refer children to family physicians (FPs) or to specialized care (4). Part of the role of PYHPs also concerns prevention and early identification of mental health problems. Mental health problems (MHPs) affect 10–20% of children and adolescents worldwide (7). MHPs are the leading cause of health-related burden in the first three decades of life (8). Half of all lifetime MHPs occur by the age of 14 years and 75% by the age of 24 years (9). To minimize the impact of MHPs, early identification is important so that adequate treatment can be provided (10).

Although PYH has an important role in the identification of MHPs as most children are regularly seen in PYH, a substantial part of MHPs are not being recognized by PYHPs (11). In order to improve the identification of child MHPs, several studies investigated the development of prediction models to identify MHPs with routine healthcare data from British and Dutch FPs. The models showed moderate predictive performances (12, 13). In the Dutch study, information regarding risk factors for MHPs related to the child's family (e.g., parental education level, parental MHPs), environment (life events) and school performance was not well-recorded in electronic health record (EHRs) of the child (13). These risk factors were important predictors for MHPs in a prospective cohort study among Dutch children from the general population in which the developed prediction model showed a good discriminative performance (14).

PYHPs gather this information regarding children and their families during check-ups and record this in the EHRs of the children, and so the information from these EHRs might potentially be useful in the identification of MHPs. For EHR data to be suitable for reuse in scientific research, the data needs to be complete, accurate and consistent (15). To our knowledge it is yet unknown how well and how complete the information is that is recorded in the EHRs. The aim of this study is to explore the usefulness of EHR data from Dutch PYH in predicting MHPs. Research questions are: what is the quality of the data and how well do they predict child MHP?

## Methods

### Study Design and Setting

A population-based cohort study was carried out using data from children aged 0–19 years visiting PYH centers of the Regional Public Health Service Hollands Midden located in the greater Leiden area, the Netherlands. The data that was anonymously extracted from the EHRs included demographics, information regarding pregnancy, family and social circumstances and information from scheduled visits and extra consultations with PYH.

The data consisted of all EHR data from 2010–2015 and all summary data from a prior electronic registration system from 2005–2010 for children born between 1994 and 2012. During the first four years of life, around 15 PYH visits are scheduled. In both primary school (children age 4–11 years) and secondary school, (children age 12–18 years) children are generally seen twice (4). The routine visit in grade 4/5 of secondary school was implemented in 2014. For all school-aged children from one routine visit [timepoint 0 (T0)], we aimed to predict the presence of MHPs during the next routine visit [timepoint 1 (T1)], thereby creating four subpopulations (Table 1). This means that for children visiting PYH at age 5/6, we used the data at the previous standard routine visit at the age of 4 years to predict mental health problems at age 5/6. We did the same for the other subpopulations.

**Table 1:** Overview of prediction moments and subpopulations.

	**T0 – Time point of measuring predictors**	**T1 – Time point of measuring outcome determinants**
Population A	Last routine visit before primary school (age ± 3–4 years)	Routine visit in grade 2 of primary school (age ± 5–6 years)
Population B	Routine visit in grade 2 of primary school (age ± 5–6 years)	Routine visit in grade 7 of primary school (age ± 10–11 years)
Population C	Routine visit in grade 7 of primary school (age ± 10–11 years)	Routine visit in grade 2 of secondary school (age ± 13–14 years)
Population D	Routine visit in grade 2 of secondary school (age ± 13–14 years)	Routine visit in grade 4 or 5 of secondary school (age ± 15–16 years)

### Outcomes

PYHPs are trained to recognize problems at an early stage. They can refer children to primary and secondary (mental) healthcare for further diagnostics or treatment. A PYHP's concern about MHPs can therefore be an early signal for child MHPs. Our main outcome was a first PYHP recorded concern for MHPs (CMHPs). We defined CMHPs 1) when PYHPs reported abnormal psychosocial functioning in the child's record, e.g., problems in making contact with others or hyperactive behavior and/or 2) when the child received extra healthcare regarding mental health (within PYH or within curative care) (Supplementary Table 1). We also performed analyses for when the outcome was only the element extra healthcare use for CMHPs as this reflects more severe MHPs.

### Determinants

Possible determinants were selected based on a PYH guideline for psychosocial problems and a systematic review regarding determinants for identified MHPs in primary care (Supplementary Table 2) (16, 17). In addition, an expert panel consisting of authors NK and MC, two FP's, a pediatrician and a PYHP, was consulted on possible determinants on based their knowledge and experience in addition to the systemic review and guidelines (13, 16). The determinants were measured up until T0. Most data was already labeled normal/abnormal. Validated cut off points, that are used in PYH, were applied to continuous data, e.g., for results of validated screening instruments Strengths and Difficulties Questionnaire (SDQ) and short indicative questionnaire for psychosocial problems among adolescents (KIVPA). The determinants number of extra healthcare visits in PYH and number of referrals were dichotomized into ≥1 yes/no. Some determinants can change over time, we then included either the first or last registered value at T0. For the other determinants we included the first known registered value. Due to sparseness of the data, we clustered closely related determinants: for example the determinant “Substance use” consisted of the items “alcohol use,” “drugs use,” “smoking,” “water pipe use,” and a more general item “substance abuse/addiction” (Supplementary Table 2). PYHPs can also include information in free text fields, due to privacy reasons we did not have access to this free text.

### Usefulness of the Data for Research

The usefulness, including completeness and validity, of the data was assessed by investigating the amount of cases (children with CMHPs), missing data and the continuity of the data, i.e., the overlap in children between populations. As children are followed in time, we expected a continuity in the data, resulting in overlapping populations.

Most determinants should either be always present in EHRs as they would always be checked during visits, e.g., length and weight, or would only be recorded in case of abnormality, e.g., smoking. The determinants SDQ and KIVPA should always be recorded, so their absence could have significance. Missingness could also mean an abnormal value and could be predictive. We therefore included a missing category in the analyses for the SDQ and KIVPA (18, 19). For the other determinants we assumed that in case a determinant was not recorded, the value of the determinant was normal (20).

### Statistical Analyses

Descriptive statistics were carried out with SPSS (version 25). If a determinant was present in <1% of the children in a subpopulation, the determinant was not included in the analysis of that subpopulation. As we aimed to predict a first recorded CMHP, we excluded children with CMHPs before or at T0. To develop prediction models for a first recorded CMHP, we performed logistic regression analyses with R (version 3.5.3) (21–24). The ability of the model to distinguish between children who are recognized with a first CMHP and those who are not (discrimination), was assessed using the c-statistic or concordance statistic (25). A c-statistic can have a value of 0 to 1, with a value of 0.5 meaning that the model is no better than predicting CMHP than random chance. The closer the value is to one the better the model. The in-sample calibration of the model was assessed by the calibration plot of actual probabilities vs. predicted probabilities. The models were internally validated using bootstrap resampling (500 bootstrap samples) and estimating shrinkage factors (26). Brier scores were calculated to assess the average prediction error: it quantifies how close predictions are to the actual outcome and can range from 0 for a perfect model to 0.25 for a non-informative model with a 50% incidence of the outcome (with a lower incidence of the outcome the maximum score for a non-informative model is lower (27, 28).

The Ethics Committee of the Leiden University Medical Centre issued a waiver of consent (G16.018).

### Role of the Funding Source

This study was supported by ZonMW, the Netherlands, Organization for Health Research and Development (grant 839110012). ZonMw did not have any role in study design, the collection, analysis, and interpretation of data, the writing of the report and the decision to submit the paper for publication.

## Results

### Usefulness of the Data for Research

This study included 26,492 children. The number of children per subpopulation ranged between 1,265 (population D) and 10,789 children (population C) (Table 2). The number of children excluded because of CMHPs ≤ T0 varied between 402 (population A) and 3,088 (population D). The overlap in children between subpopulations was low and the number of CMHPs varied greatly between populations. Population C had a high number of CMHPs, much higher than the other subpopulations, which might be largely explained by limited overlap in children between population B and C. We assumed that population C contained not only incident cases but also prevalent cases of CMHPs, which could not be excluded since no information of these children from before the age of 10 was present. For population B the overlap with previous years was also small, but in that population it concerned data from the pre-school period. During the pre-school period MHPs are less frequently identified and therefore the CMHPs in population B were more likely to refer to incident CMHPs (29, 30).

**Table 2:** Overview of subpopulation and outcomes.

**Study subpopulation**	**A**	**B**	**C**	**D**
Number of children included (*n*)	10,146	6,606	10,779	1,265
Number of children excluded as CMHPs < T0 (*n*)	402	2494	1,599	3,088
Overlap in children with previous population (%)	Not applicable	0.3%	13.7%	64.7%
CMHPs, % (*n*)	35.8 (3,628)	8.5 (564)	57.8 (6,276)	7.1 (90)
(a) Extra healthcare use only, % (*n*)	2.8 (283)	5.0 (327)	3.3 (362)	3.8 (48)
(b) Abnormal mental health functioning only, % (*n*)	25.0 (2,538)	1.0 (63)	36.5 (3,962)	0.9 (12)
(c) Both extra healthcare use and abnormal mental health functioning, % (*n*)	8.0 (807)	2.6 (174)	18.0 (1,952)	2.3 (30)
Extra healthcare use, total of (a) and (c)	10.7 (1,090)	7.6 (502)	21.4 (1,343)	6.2 (78)

*CMHPs, concerns for mental health problems*.

Since our aim was to predict incident CMHPs and different determinants can play a role in incident or prevalent cases, we excluded population C from further analyses.

The amount of missing data from the determinants ranged from 4.4 to 100%, a large number of determinants had missing data for over 80% of the children (Supplementary Table 3).

### Prediction of a First Concern of Mental Health Problems

#### Population A

Population A consisted of 10,146 children aged 3–4 years of which 3,628 children (35.8%) had a first recorded CMHPs during the next routine visit at age 5–6 years (Table 2). Determinants for CMHPs were male gender, developmental problems, family history of MHPs, extra healthcare visit in PYH and a negative balance in protective factors and risk factors for a child's healthy development (Tables 3, 4). A non-spontaneous birth was associated with a decreased risk of CMHPs. Extra healthcare use for CMHPs was recorded in 10.7% of all children. Family history of MHPs and a negative balance were associated with this extra healthcare use (Table 5). In addition, children with an extra healthcare visit in PYH or environmental stressors were less likely to receive extra healthcare use for CMHPs.

**Table 3:** Baseline characteristics study population.

**Characteristics^a^**	**Population A *N* = 10,146 % (*n*)**	**Population B *N* = 6,606 % (*n*)**	**Population C *N* = 10,789 % (*n*)**	**Population D *N* = 1,265 % (*n*)**
CMHPs	35.8 (3,628)	5.8 (564)	58.2 (6,276)	7.1 (90)
Age in years (mean, sd)	3.96 (0.14)	5,85 (0,46)	10,96 (0,52)	13,88 (0,53)
Male gender	50.3 (5,103)	48.1 (3,176)	49.5 (5,339)	48.8 (617)
Ethnicity	0.0 (0)	0,6 (42)	0.0 (0)	4.4 (56)
Premature	5.1 (518)	0.0 (0)	0.4 (41)	0.9 (12)
Neonatal problems	1.1 (116)	2.7 (181)	0.4 (48)	0.2 (3)
Non-spontaneous birth	9.0 (909)	0.0 (2)	1.1 (114)	3.9 (49)
Developmental problems	3.0 (304)	2.1 (136)	0.5 (49)	0.9 (11)
Incontinence	NA	0.6 (41)	0.7 (76)	0.9 (12)
Excessive crying	0.1 (12)	NA	NA	NA
Sleeping problems	0.2 (16)	0.1 (8)	0.0 (0)	0.1 (1)
Eating problem	0.0 (0)	0.2 (12)	0.0 (4)	0.0 (0)
Overweight	8.6 (871)	2.5 (167)	7.4 (802)	13.0 (164)
Underweight	14.2 (1,442)	4.8 (320)	4.6 (497)	10.4 (132)
School problem	0.1 (12)	1.5 (102)	0.5 (54)	0.9 (12)
Secondary school level low	NA	NA	15.1 (1,628)	31.9 (404)
Secondary school level high	NA	NA	0.0 (0)	28.7 (363)
Secondary school level other	NA	NA	0.0 (3)	0.6 (8)
Bullying/being bullied	NA	0.0 (2)	0.0 (4)	0.2 (2)
Low self-confidence/resilience	0.1 (13)	0.1 (8)	0.0 (0)	0.0 (0)
Member of hobby/music club	NA	0.0 (1)	96.4 (10,405)	0.0 (0)
Insufficient physical exercise	0.0 (0)	0.0 (0)	1.0 (103)	0.2 (3)
Substance use	NA	NA	0.1 (8)	0.0 (0)
High technology use	0.0 (0)	0.0 (0)	6.8 (729)	0.4 (5)
SDQ borderline	NA	3.0 (197)	6.3 (682)	4.8 (61)
SDQ increased	NA	1.4 (95)	4.1 (447)	2.1 (27)
SDQ missing	NA	32.1 (2,121)	40.4 (4,364)	545 (43.1)
KIVPA increased	NA	NA	NA	6.2 (78)
KIVPA missing	NA	NA	NA	4.6 (58)
Under treatment	0.0 (0)	15.7 (1,035)	2.8 (306)	4.0 (51)
Total referral	6.1 (614)	0.1 (5)	0.1 (6)	0.7 (9)
Extra healthcare visit	33.5 (3,398)	9.4 (621)	11.2 (1,208)	26.1 (330)
Life events	4.4 (442)	9.8 (648)	6.6 (708)	7.5 (95)
Family related
Family history of MHPs	2.1 (217)	1.8 (117)	0.5 (53)	0.9 (11)
Chronic illness parent	3.1 (315)	0.3 (21)	0.8 (81)	0.7 (9)
Risk factor parents	3.3 (334)	11.3 (749)	8.1 (870)	7.6 (96)
Prenatal risk factors	5.0 (503)	0.0 (0)	0.7 (75)	2.2 (28)
Non-traditional family composition	1.4 (146)	0.7 (49)	0.7 (79)	11.8 (149)
Negative balance	2.5 (253)	0.2 (10)	NA	NA
Little confidence in parenting skills	0,1 (15)	1.0 (66)	0.1 (14)	0.2 (2)
Environmental stressors	7.9 (799)	0.6 (38)	2.7 (287)	6.0 (76)
Nr of Contact moments available (median, IQR)	6 (5)	4 (2)	3 (2)	4 (2)

a *Determinants were excluded from analysis when the determinant was present in <1% of the children of a population. The determinant incontinence is excluded at study population A because before primary school (T0) incontinence is considered normal. CMHPs, concerns for mental health problems; NA, not applicable; SDQ, strengths and difficulties questionnaire; KIVPA, short indicative questionnaire for psychosocial problems among adolescents; MHPs, mental health problems*.

**Table 4:** Results of logistic regression analysis for a first recorded concern for MHPs.

**Characteristics**	**Study population A** ***N*** **=** **10,146 nr of events 3,628**			**Study population B** ***N*** **=** **6,606 nr of events 564**			**Study population D** ***N*** **=** **1,265 nr of events 90**		
	**Coefficient**	**OR**	**95% CI**	**Coefficient**	**OR**	**95% CI**	**Coefficient**	**OR**	**95% CI**
Intercept	−0.91			−2.50			−1.90		
Male gender	0.31	1.30	1.20–1.41	0.14	1.07	0.90–1.28	−0.12	0.48	0.31–0.75
Ethnicity							−0.39	0.30	0.09–1.04
Premature	0.19	1.14	0.95–1.37						
Neonatal problems	0.02	0.95	0.64–1.42	−0.18	0.76	0.43–1.36			
Non-spontaneous birth	−0.17	0.77	0.66–0.90						
Developmental problems	0.46	1.53	1.21–1.93	0.69	1.94	1.22–3.09			
Overweight	0.20	1.15	0.99–1.33	−0.24	0.71	0.39–1.30	−0.01	0.59	0.30–1.09
Underweight	−0.02	0.91	0.81–1.03	0.01	0.93	0.61–1.40	−0.20	0.42	0.19–0.94
Negative weight perception
School problem				0.72	2.02	1.21–3.38			
Secondary school level low							0.19	0.81	0.47–1.41
Secondary school level high							0.16	0.77	0.44–1.37
SDQ borderline				1.20	3.37	2.17–5.27	0.41	1.18	0.45–3.07
SDQ increased				0.06	0.99	0.53–1.84	0.01	0.59	0.15–2.32
SDQ missing				0.01	0.93	0.77–1.14	0.20	0.83	0.51–1.36
KIVPA increased							0.71	1.95	1.00–3.80
KIVPA missing							0.70	1.95	0.89–4.28
Under treatment				0.00	0.92	0.72–1.17	−0.47	0.26	0.07–0.92
Total referral	0.06	0.99	0.83–1.18						
Extra healthcare visit	0.16	1.11	1.01–1.22	0.07	1.00	0.77–1.33	0.35	1.06	0.64–1.75
Life events	0.26	1.22	1.00–1.50	0.70	1.97	1.55–2.49	0.71	1.98	0.96–4.09
Family history of MHPs	0.50	1.60	1.21–2.12	0.55	1.67	1.00–2.79			
Chronic illness parent	−0.08	0.85	0.67–1.09						
Risk factor parents	0.03	0.96	0.76–1.22	0.21	1.16	0.90–1.50	0.13	0.73	0.32–1.64
Prenatal risk factors	0.04	0.97	0.79–1.18				−0.67	0.19	0.02–1.49
Non-traditional family composition	0.06	0.99	0.69–1.41				0.01	0.59	0.30–1.17
Negative balance	0.77	2.12	1.64–2.75						
Little confidence in parenting skills				0.66	1.88	1.03–3.44			
Environmental stressors	0.12	1.06	0.91–1.23				−0.58	0.23	0.07–0.81
C-statistic corrected	0.54			0.57			0.40		
Shrinkage factor B=500	0.93			0.92			0.59		
Brier score	0.22			0.08			0.06		

*SDQ, strengths and difficulties questionnaire; KIVPA, short indicative questionnaire for psychosocial problems among adolescents; MHPs, mental health problems*.

**Table 5:** Results of logistic regression analysis for the first recorded extra healthcare use for concerns for MHPs.

**Characteristics**	**Study population A** ***N*** **=** **10,146 nr of events 1,090**			**Study population B** ***N*** **=** **6,606 nr of events 502**			**Study population D** ***N*** **=** **1,265 nr of events 78**		
	**Coefficient**	**OR**	**95% CI**	**Coefficient**	**OR**	**95% CI**	**Coefficient**	**OR**	**95% CI**
Intercept	−1.95			−2.58			−1.95		
Male gender	0.24	1.12	0.98–1.27	0.14	1.06	0.88–1.28	−0.20	0.41	0.25–0.66
Ethnicity							−0.33	0.32	0.09–1.12
Premature	0.05	0.79	0.59–1.07						
Neonatal problems	0.32	1.22	0.70–2.14	−0.12	0.79	0.43–1.44			
Non-spontaneous birth	−0.15	0.71	0.56–0.90						
Developmental problems	0.28	1.17	0.84–1.63	0.76	2.10	1.31–3.36			
Overweight	0.17	1.03	0.83–1.28	−0.32	0.64	0.33–1.23	−0.11	0.47	0.23–0.97
Underweight	0.00	0.84	0.70–1.01	0.01	0.91	0.59–1.40	−0.23	0.39	0.16–0.91
School problem				0.23	1.17	0.63–2.18			
Secondary school level low							0.18	0.79	0.43–1.44
Secondary school level high							0.23	0.86	0.47–1.58
SDQ borderline				1.12	3.10	1.95–4.94	0.47	1.31	0.48–3.59
SDQ increased				0.20	1.12	0.59–2.14	−0.18	0.41	0.08–2.05
SDQ missing				−0.03	1.88	1.52–2.31	0.20	0.82	0.48–1.38
KIVPA							0.65	1.79	0.88–3.64
KIVPA missing							0.72	2.02	0.88–4.64
Under treatment				0.02	0.92	0.72–1.19	−0.39	0.29	0.08–1.04
Total referral	0.12	0.97	0.75–1.26						
Extra healthcare visit	−0.03	0.81	0.70–0.94	0.09	1.01	0.74–1.36	0.33	1.03	0.60–1.75
Life events	0.20	1.06	0.80–1.41	0.72	2.01	1.57–2.56	0.81	2.35	1.12–4.93
Family history of MHPs	0.66	1.85	1.31–2.62	0.53	1.62	0.95–2.76			
Chronic illness parent	−0.15	0.70	0.48–1.03						
Risk factor parents	0.29	1.18	0.86–1.62	0.24	1.18	0.90–1.54	0.24	0.88	0.38–2.00
Prenatal risk factors	−0.10	0.75	0.55–1.02				−0.66	0.18	0.02–1.48
Non-traditional family composition	0.20	1.07	0.66–1.72				0.03	0.60	0.29–1.23
Negative balance	0.48	1.49	1.07–2.07						
Little confidence in parenting skills				0.60	1.76	0.94–3.30			
Environmental stressors	−0.28	0.60	0.46–0.78				−0.48	0.25	0.07–0.90
C-statistic corrected	0.48			0.57			0.41		
Shrinkage factor B = 500	0.84			0.91			0.57		
Brier score	0.10			0.07			0.06		

*SDQ, strengths and difficulties questionnaire; KIVPA, short indicative questionnaire for psychosocial problems among adolescents; MHPs, mental health problems*.

#### Population B

In 564 (8.5%) children aged 5–6 years, a first recorded CMHPs was found during the next visit at age 10–11 years (population B). Extra healthcare use for CMHPs was recorded in 502 (7.6%) of children. The determinants developmental problems, school problems, SDQ borderline test results, life events and parents' little confidence in parenting skills were associated with an increased risk of CMHPs. Other determinants were not associated with CMHPs. The analysis with extra healthcare use for CMHPs showed similar results apart from school problems and little confidence in parenting skills both showing no association with the outcome.

#### Population D

Population D included 1,265 children aged 13–14 years of which 90 (7.0%) had a first recorded CMHPs at age 15–16 years. Extra healthcare use for CMHPs was recorded in 78 (6.2%) children. Male gender, being underweight, being under treatment for any reason and environmental stressors were associated with a decreased risk of CMHPs. An increased KIVPA score was associated with an increased risk of CMHPs. Regarding the outcome extra healthcare use for CMHPs results were similar, apart from extra healthcare visit within PYH, being under treatment and environmental stressors not being associated with extra healthcare use for CMHPs. In addition, children being overweight or underweight were less likely to receive extra healthcare use for CMHPs. Other determinants, including increased SDQ scores were not associated with both outcomes.

## Model Performance

The models' discriminatory accuracies for a first recorded CMHPs were low with corrected c-statistics of, respectively, 0.54, 0.57, and 0.40 for populations A, B, and D. Internal validation for the models showed shrinkage factors of 0.93 for population A, 0.82 for population B and 0.54 for population D and varying calibration (Supplementary Figure 1). The Briers scores varied from 0.07/0.08 (population D and B) to 0.22 (population A). Regarding the models for extra healthcare use for CMHPs, the c-statistics were slightly lower with a range of 0.41–0.57. Shrinkage factors and Brier scores were similar.

## Discussion

In this population-based cohort study we explored the usefulness of routine healthcare data from Dutch PYH in predicting MHPs. The usefulness of the data was suboptimal as the number of cases differed greatly between subpopulations, a substantial part of the data was missing and the continuity of the data, i.e., following children for a longer time period resulting in overlapping populations, was much less than expected. We aimed to develop prediction models in school-aged children visiting PYH that would predict first concerns for MHPs during the next routine check-up in PYH. Unfortunately, the discriminatory performances of the models were poor and the models in their current form appeared not to be useful in the early identification of MHPs.

The use of data from routine EHRs has become increasingly popular over the past years, also for policy purposes (31). To our knowledge this is the first study exploring the usefulness of EHRs from Dutch PYH in predicting child MHPs. Our population-based cohort study reflects Dutch routine PYH and gives an insight into the current state of the electronic healthcare registration of PYH. Although we expected that there would be a continuity in the data as we aimed to follow children for a longer time, we observed little overlap between the different subpopulations. Our time window of 2005–2015 and the fact that children can go to secondary schools outside the region, meaning they are monitored by a different regional PYH of which we did not possess data, might play a role, but we expect other (technical) reasons we are not yet aware of to also play a role: such as changes in registration systems (e.g., the change from paper to digital in 2010) in which data from the old system needed to be migrated to the new system. This meant that it was difficult to exclude prevalent CMHP cases from successive populations. In population C for instance, 58% of the children were found to have CMHPs, much higher than expected according to literature (7, 17). Population D was small, as the timepoint 1 visit was only implemented in 2014, this resulted in less stable models.

The electronic system PYHPs use to record findings from clinical care is technically built in such a manner that important information from previous consultations should remain present in the system. For instance, information on ethnicity, pregnancy and birth weight would still be present during visits in primary school. However, in our extracted data, this was not always the case, resulting in substantial missing data for many of these unchangeable determinants. We do not think missing data played a large role in our outcome, as (extra healthcare use for) CMHPs when present, would be a specific finding PYHPs would register as it is part of the basic tasks of PYH. Missing data in routine healthcare datasets are a known problem (20). One way to reduce the effect of missing data is imputation. However within routine healthcare data, missing data is seldom solely missing at random, which means you have to carefully choose your method of imputation and choosing not to impute might even be the better option (18, 20). In this study, we applied the commonly used assumption that a missing value would indicate a negative value, or in other words “if it is not mentioned, it is not there” (20) for most determinants. Given the large amount of missing data, we question whether this assumption still holds as prevalence rates of determinants such as family MHP or smoking were lower than expected from literature (32, 33). For determinants SDQ and KIVPA, which should be filled out by all parents of primary school students and adolescents in secondary schools prior to visiting a PYH and is registered standardly in the registration system, we included a missing category as missing data could refer to parents not being able (illiteracy, non-dutch) or wanting to fill-out the questionnaire which could be predictive. This did not result in better performing models. Our study was the first study examining routine healthcare data from preventive youth healthcare with regards to child MHP identification. Such medical registries were originally built to assist healthcare professionals in daily practice, they were not built for research purposes. It is known that it takes time to improve medical registries in such way that they can be better used for research purposes (34).

Several strategies to improve the quality of electronic healthcare data are suggested in the literature, which could also apply to the electronic health data of the PYH (20). Training professionals in accurate recording has proven to enhance the quality of registered data in primary care (34). Another suggested strategy is the implementation of information from external sources (20). Part of the missing data in this study, e.g., information regarding parental educational level, financial problems, and information regarding birth and pregnancy, could possibly be improved by linking data from Statistics Netherlands and the Dutch Perinatal Registry (35, 36). Another solution might be the implementation of short electronic questionnaires prior to scheduled visits in which parents fill out relevant information with an automatic upload into the child's EHR. Or, like the Dutch Perinatal Registry, create a national dataset with key information which is gathered in a standardized way. An even more advanced option would be a shared digital record in which parents and PYHPs can both record information. PYHPs can also include relevant information regarding determinants in free text which we did not have in our extraction due to privacy reasons. We recommend to repeat this study with improved data and to investigate the usefulness of free text, for instance with natural language processing techniques (37).

The developed models in this study had a poor predictive performance, however we found that some known risk factors for MHPs had a predictive value. In addition, several determinants such as previous extra PYH visits and school problems, were associated with CMHPs, but not with extra healthcare use for CMHPs, meaning that PYHPs have concerns and monitor, but do not opt for extra care. Determinants like environmental stressors and parental concerns regarding parenting skills were even associated with a decreased risk of extra healthcare use for CMHPs. This could indicate that PYHs have concerns regarding the child's environment rather than regarding MHPs of the child itself. One can imagine that PYPHs in this case would use preventive interventions aimed at the child's environment, like Triple P, which could affect children positively (38). Regarding life events, our study suggests that PYHPs are less likely to monitor as life events in the older age groups were associated with an increased risk of (extra healthcare use for) CMHPs. In addition, because our outcome measurement CMHPs is based on the judgement of PYPHs and is not an objective measurement, this makes predicting CMHPs more difficult to begin with.

Increased SDQ-scores for psychosocial problems had limited prognostic value, whereas borderline increased SDQ-scores were associated with an increased risk of (extra healthcare use for) CMHPs. This can be explained by the fact that SDQ-scores were measured at T0. We saw that children with increased SDQ-scores at T0 were more likely to have registered CMHPs at the same T0 and would therefore be excluded from our study. This was less likely for the borderline scores. Another explanation can be that screening instruments are not always predictive for PYHPs' actions and concerns. Mieloo and colleagues found that when using a screening instrument, 38% of the children with an increased score on that instrument were registered as such by the PYHP and 22% of the children with an increased score were referred for extra care (39). It would be interesting to investigate what PYHPs do with increased SDQ-scores, also during later visits.

In contrast to our findings, a prospective cohort study in the Dutch general population which developed models that estimated the risk of MHPs in adolescents showed a good performance (14). In this study, information on determinants was collected via questionnaires that were sent to the parents. Important determinants for MHPs were, amongst others, maternal educational level, family history of psychopathology and environmental stressors such as frequently moving house, severe disease or death in the family, and parental divorce (14). A lot of these determinants did not show a positive association with CMHPs in our study although they are known risk factors for MHPs (16). A possible explanation for this might be the high number of missing values in this study.

We are aware that the data we used in this study is specific to the Dutch healthcare system and the registration used in this particular region, and we expect the generalizability of our findings to be limited in other settings. However, many countries do have a form a preventive youth healthcare or well-child clinics, that monitor a child's healthy development in some way (1–3). In addition, validated mental health screening instruments are widely used (40).

Depending on the type of preventive youth healthcare and digital registration used, we would recommend adapting our current approach to different settings and available routine healthcare data to explore the possibilities of digital information from preventive youth healthcare for the early identification of child MHPs.

## Conclusion

In conclusion, this study explored the usefulness of data acquired from EHRs from Dutch PYH in estimating the risk of mental health problems in children. The data quality was sub-optimal and the developed prediction models showed poor performances. When data quality can be improved by facilitating accurate recording and increasing the proportion of data that can be entered through forms of structured input, EHR data from PYH is likely to be valuable in its contribution to the timely recognition of child MHPs.

## Data Availability Statement

The datasets presented in this article are not readily available because of the nature of the included data. Requests to access the datasets should be directed to n.r.koning@lumc.nl.

## Author Contributions

NK conceptualized and designed the study, carried out the analyses, and drafted the initial manuscript and revised the manuscript. FB, MC, and MN conceptualized and designed the study, had access to the data and supervised the analyses, and critically reviewed and revised the manuscript. AB and SC carried out the analyses and revised the manuscript. IP, NL, and DD conceptualized the study, provided the data, participated in the interpretation of the data, and reviewed and revised the manuscript for important intellectual content. All authors approved the final manuscript as submitted and agree to be accountable for all aspects of the work.

## Conflict of Interest

The authors declare that the research was conducted in the absence of any commercial or financial relationships that could be construed as a potential conflict of interest.

## Abbreviations

CMHPs: concern for mental health problems
EHR: electronic health record
FPs: family physicians
KIVPA: short indicative questionnaire for psychosocial problems among adolescents
MHPs: mental health problems
PYH: preventive youth healthcare
PYHPs: preventive youth healthcare professionals
SDQ: strengths and difficulties questionnaire
T0: timepoint 0
T1: timepoint 1.
