# Supplementary material for: The Usefulness of Electronic Health Records From Preventive Youth Healthcare in the Recognition of Child Mental Health Problems
Source: Front Public Health. 2021 May 31;9:658240. doi: 10.3389/fpubh.2021.658240 (PMC8202822; doi:10.3389/fpubh.2021.658240)
Supplement: Supplementary file 1 [file Table_1.DOCX]

**Supplement tables and figure**

-Supplement table 1: Definition of outcome concerns for mental health problems (CMHPs)
-Supplement table 2: Definition of determinants
-Supplement table 3: Missing data of determinants per subpopulation
-Supplement figure 1: Calibration plots for concerns for mental health problems (CMHP) (A, B, C) and extra healthcare use for CMHP (D, E, F)

**Supplement table 1: Definition of outcome concerns for mental health problems (CMHPs)**

| **Outcome** | **Definition** |
| --- | --- |
| Extra healthcare use for MHP  (>1 components) | >1 referral  to a mental health specialist with indication mental health |
|  | >1 consultation  with a mental health specialist with indication mental health |
|  | Extra healthcare use between standard visits with indication mental health |
|  | >1 intervention for mental health:  -Triple P^a^ level 3 or higher and tip sheets (fears in children, stealing, dealing with fear or depression) |
| Finding of abnormal mental health functioning  (>1 components) | Atypical mental health  functioning (single examination by a community pediatrician) |
|  | >1 abnormal specific mental health functioning recorded |

^a^Triple P = “Positive Parenting Program”, a multilevel program to support parents with children aged 0-16 years with the aim of reducing the prevalence of MHP, emotional and behavioral problems in children by teaching parents parenting skills. The multilevel program has 5 intensity levels, with level 5 as the most intensive program.(Sanders, 2008; Schappin et al., 2017)
MHP = mental health problem

**Supplement table 2: Definition of determinants**

| **Determinant** | **Definition^a^** | **Timing: first or last recorded measurement ≤T0** |
| --- | --- | --- |
| Gender | First recorded gender in electrical child record | First |
| Premature | Pregnancy duration <37 weeks or 259 days | First |
| Ethnicity | Immigrant/refugee  Country of birth of ≥1 parent is other than the Netherlands or West-Europe (e.g. Suriname Dutch Antilles, Turkey, Morocco, Eastern Europe, other non-Western countries) | First |
| Non spontaneous birth | Cesarean section, vaginal birth with forceps or vacuum extraction | First |
| Delay in development | General developmental delay and/or speech and language delay at age 7 years and older | First |
| Incontinence for urine and defecation | Incontinent for urine and defecation at age 4 years and older | Last |
| Excessive crying | Excessive crying, more than a short phase | First |
| Sleeping  problems | Sleeping problems | Last |
| Eating Problem | Eating Problem | Last |
| Overweight | BMI classified as overweight or obese according to international age and gender specific standards(Cole et al., 2000; Kist-van Holthe et al., 2012) | T0 |
| Underweight | BMI classified as underweight according to international age and gender specific standards(Cole et al., 2000; Kist-van Holthe et al., 2012) | T0 |
| Negative weight perception | Negative perception of own weight (too light or too heavy) | T0 |
| School problem | Any reported problems in school e.g. dyslexia, difficulty focusing, motivation problems, absence or declining school performance | First |
| Secondary school education level | Secondary school education level divided into 3 categories according to the Dutch school system:  -low: VMBO or lower -middle: HAVO (reference category) -high: VWO  -Other: in case of special education or no education; HAVO is reference category)  When combined education levels were recorded, the lowest level was chosen, e.g. HAVO for HAVO/VWO | Last |
| Bullying/being bullied | Bullying or being bullied | First |
| Bad relationship with at least one parent | Bad relationship with at least one parent | Last |
| Low self-confidence/ resilience | Low self-confidence/ resilience | Last |
| Self-harm | Auto mutilation or suicidal thoughts | First |
| Female genital mutilation | Female genital mutilation | First |
| Unemployment or financial distress of the child | Unemployment or financial distress of the child | Last |
| Member of hobby of music club | Member of a hobby or music club | Last |
| Insufficient physical exercise | Less than one hour of exercise a day and/or not enough physical exercise according to the EMOVO^b^ questionnaire: cycling or walking to school or an internship less than 1 day a week | Last |
| Substance use | Alcohol use: at least once a week an alcoholic consumption | Last |
|  | Drugs use: using or ever used hard drugs or soft drugs | Last |
|  | Smoking: smoking or ever smoked | Last |
|  | Water pipe use, at least once a week | Last |
|  | Substance abuse/addiction  (sum of the use of alcohol, drugs, smoking, waterpipe) and additional element | Last |
| Excessive Energy drink consumption | Energy drink abuse/addiction, consumes more than 1 energy drink a day | Last |
| Technology use | Gaming: more than 3 days a week | Last |
|  | Social media use more than 3 days a week | Last |
|  | Screen use on average daily over 2 hours of television or computer use | Last |
| SD border area^c^ | SDQ total score between normal and increased limits (border area)  -total score 3 years: 9-11  -total score 4-7 years: 11-14  -total score 8-14 years: 11-13  -total score 15-19 years: 13-15 | Last |
| SDQ increased^c^ | Increased SDQ total score  -total score 3 years: 12-40  -total score 4-7 years: 15-40  -total score 8-14 years: 14-40  -total score 15-19 years:16-40 | Last |
| KIVPA^d^ | Increased KIVPA score ≥6 is an indication for consultation with PYHP. Maximum is 25 points | Last |
| Under treatment | Already perceiving any form of treatment | Last |
| Medical referral | Medical referral | until T0 |
| Paramedical referral | Referral to speech therapist, dietician of physical therapist | until T0 |
| Other referral | All referrals except medical or paramedical referrals, e.g. parenting support, home counseling, program for overweight children | until T0 |
| Total referral | Sum of all above referrals |  |
| Extra healthcare visit | Extra healthcare visit in preventive youth healthcare on top of standard visits, excluding visits for MHP and vaccinations | Until T0 |
| Life events | Looked after children (children whom are (temporarily) in a foster family, living in an  institution only when parents cannot take care of the child or custody by other person than family member | First |
|  | Conflicts within household/hostile  atmosphere | First |
|  | Death of parent(s), sibling or other significant person. | First |
|  | Victim of violence/abuse | First |
|  | Divorce parent(s) or abandonment by parent | First |
|  | Adoption | First |
|  | Immigrant/refugee | First |
| Mental health in family history | Parents with any mental health problem | First |
|  | Siblings with any mental health problem | First |
| Chronic Illness parent | Parent with chronic illness | First |
| Risk factors parents | Parent victim of abuse in youth | Last |
|  | Start of parenting support program “Stevig ouderschap”, which helps parent(s) with a difficult start, for example due to the medical history of the parent or child, personal problems, insufficient supportive environment | Last |
|  | Little support from social network parents | Last |
|  | Unemployment or financial distress parents | Last |
|  | Both parents with low level of completed education according to the International Standard Classification of Education(Statistics, 2012): no, primary or lower secondary education | Last |
| Prenatal risk factors | Substance abuse (smoking, alcohol or drugs) of the mother during pregnancy | First |
|  | Young parenthood: 1 or more parent <20 years old at birth | First |
|  | Complications during pregnancy (IVF/ICSI, blood loss in 1st or 2nd trimester,  hypertension, diabetes) | First |
|  | Medication use during pregnancy (all  prescribed oral medication to mother during  pregnancy) | First |
| Non-traditional family composition | All non-two parent family compositions, e.g. co-parent family composition, steph-parent family composition | Last |
| Negative balance | Based on the model of Bakker (Akkerman et al., 2012) which combines different protective factors and risk  factors for a child's healthy development on micro- meso- and macro level | Last |
| Parental concerns | Parents are concerned about their child on any aspect | Last |
| Little confidence parenting skills, non-optimal parenting skills | Little confidence in parenting skills and/or parents with problems with parenting according to triple P multilevel program with level 3 or higher | First |
| Environmental stressors | Long hospital admittance child | Last |
|  | Long hospital admittance sibling | Last |
|  | Expansion in the family by sister, brother or step-parent, step-brother or step-sister | Last |
|  | Move/migration | Last |
|  | Conflict outside of household | Last |

**^a^**All definitions of the determinants are binary (yes/no), information regarding developmental delay, incontinence, school problems including bullying, substance use, mental health problem (MHP) screening tools Strengths and difficulties questionnaire (SDQ) and short indicative questionnaire for psychosocial problems among adolescents (KIVPA), life events, family MHPs and parental educational level was available from the period 2005-2015, information regarding the other predictors was available from the period 2010-2015.
^b^EMOVO = a digital questionnaire of Dutch preventive youth healthcare (PYH) to monitor the health and well-being of second and fourth graders of secondary school(De Nooijer and De Vries, 2007)
^c^Strengths and difficulties questionnaire (SDQ) = short screening questionnaire to screen for MHP in children 2-17 years old(Van Widenfelt et al., 2003)
^d^KIVPA = a short indicative questionnaire for psychosocial problems among adolescents(Reijneveld et al., 2003)

**Supplement table 3 Missing data of determinants per subpopulation**

| **Characteristics** | **Population A N=29504** | | **Population B N= 6606** | | **Population C N= 10789** | | **Population D N=1265** | |
| --- | --- | --- | --- | --- | --- | --- | --- | --- |
|  | **% (n)** | **% missing data** | **% (n)** | **% missing data** | **% (n)** | **% missing data** | **% (n)** | **% missing data** |
| **Age in years (mean, std)** | 3.96 (0.14) |  | 5,85 (0,46) |  | 10,96 (0,52) |  | 13,88 (0,53) |  |
| **Male gender** | 50.3 (5103) | 0.0 | 48.1 (3176) | 0.0 | 49.5 (5339) | 0.0 | 48.8 (617) | 0.0 |
| **Ethnicity** | 0.0 (0) | 100 | 0,6 (42) | 96.7 | 0.0 (0) | 100 | 4.4 (56) | 80.6 |
| **Premature** | 5.1 (518) | 27.5 | 0.0 (0) | 99.8 | 0.4 (41) | 94.6 | 0.9 (12) | 81.0 |
| **Neonatal problems** | 1.1 (116) | 70.4 | 2.7 (181) | 93.3 | 0.4 (48) | 22.1 | 0.2 (3) | 63.2 |
| **Non-spontaneous birth** | 9.0 (909) | 72.4 | 0.0 (2) | 99.9 | 1.1 (114) | 95.3 | 3.9 (49) | 85.4 |
| **Developmental problems** | 3.0 (304) | 43.4 | 2.1 (136) | 95.3 | 0.5 (49) | 22.7 | 0.9 (11) | 37.8 |
| **Incontinence** | NA | NA | 0.6 (41) | 94.7 | 0.7 (76) | 15.6 | 0.9 (12) | 37.8 |
| **Excessive crying** | 0.1 (12) | 99.6 | NA | NA | NA | NA | NA | NA |
| **Sleeping problems** | 0.2 (16) | 99.8 | 0.1 (8) | 6.9 | 0.0 (0) | 25.4 | 0.1 (1) | 45.6 |
| **Eating problem** | 0.0 (0) | 100 | 0.2 (12) | 6.9 | 0.0 (4) | 25.4 | 0.0 (0) | 45.6 |
| **Overweight** | 8.6 (871) | 0.4 | 2.5 (167) | 74.2 | 7.4 (802) | 50.7 | 13.0 (164) | 1.6 |
| **Underweight** | 14.2 (1442) | 0.4 | 4.8 (320) | 74.2 | 4.6 (497) | 50.7 | 10.4 (132) | 1.6 |
| **School problem** | 0.1 (12) | 99.6 | 1.5 (102) | 6.9 | 0.5 ( 54) | 25.4 | 0.9 (12) | 45.5 |
| **Secondary school level low** | NA | NA | NA | NA | 15.1 (1628) | NA | 31.9 (404) | NA |
| **Secondary school level high** | NA | NA | NA | NA | 0.0 (0) | NA | 28.7 (363) | NA |
| **Secondary school level other** | NA | NA | NA | NA | 0.0 (3) | NA | 0.6 (8) | NA |
| **Bullying/being bullied** | NA | NA | 0.0 (2) | 6.4 | 0.0 (4) | 24.3 | 0.2 (2) | 43.6 |
| **Low self-confidence/resilience** | 0.1 (13) | 99.6 | 0.1 (8) | 6.9 | 0.0 (0) | 25.4 | 0.0 (0) | 45.5 |
| **Member of hobby/music club** | NA | NA | 0.0 (1) | 100 | 96.4 (10405) | 0.0 | NA | NA |
| **Insufficient physical exercise** | 0.0 (0) | 100 | 0.0 (0) | 100 | 1.0 (103) | 86.1 | 0.2 (3) | 99.1 |
| **Substance use** | NA | NA | NA | NA | 0.1 (8) | 17.0 | 0.0 (0) | 44.8 |
| **High technology use** | 0.0 (0) | 100 | 0.0 (0) | 100 | 6.8 (729) | 85.8 | 0.4 (5) | 99.0 |
| **SDQ border area** | NA | NA | 3.0 (197) | 32.1 | 6.3 (682) | 40.4 | 4.8 (61) | 43.1 |
| **SDQ increased** | NA | NA | 1.4 (95) | 32.1 | 4.1 (447) | 40.4 | 2.1 (27) | 43.1 |
| **KIVPA** | NA | NA | NA | NA | NA | NA | 6.2 (78) | 4.6 |
| **Under treatment** | 0.0 (0) | 100 | 15.7 (1035) | 84.3 | 2.8 (306) | 97.2 | 4.0 (51) | 96.0 |
| **Total referral** | 6.1 (614) | NA | 0.1 (5) | NA | 0.1 (6) | NA | 0.7 (9) | NA |
| **Extra healthcare visit** | 33.5 (3398) | NA | 9.4 (621) | NA | 11.2 (1208) | NA | 26.1 (330) | NA |
| **Life events** | 4.4 (442) | 85.5 | 9.8 (648) | 5.1 | 6.6 (708) | 20.4 | 7.5 (95) | 37.4 |
| **Family history of MHP** | 2.1 (217) | 79.4 | 1.8 (117) | 4.4 | 0.5 (53) | 20.6 | 0.9 (11) | 40.2 |
| **Chronic illness parent** | 3.1 (315) | 79.7 | 0.3 (21) | 97.4 | 0.8 (81) | 91.7 | 0.7 (9) | 89.6 |
| **Risk factor parents** | 3.3 (334) | 64.2 | 11.3 (749) | 5.1 | 8.1 (870) | 46.3 | 7.6 (96) | 53.8 |
| **Prenatal risk factors** | 5.0 (503) | 82.0 | 0.0 (0) | 96.1 | 0.7 (75) | 97.2 | 2.2 (28) | 71.9 |
| **Non-traditional family composition** | 1.4 (146) | 72.0 | 0.7 (49) | 93.2 | 0.7 (79) | 94.3 | 11.8 (149) | 15.6 |
| **Negative balance** | 2.5 (253) | 51.0 | 0.2 (10) | 96.1 | NA | NA | NA | NA |
| **Little confidence in parenting skills** | 0,1 (15) | 88.8 | 1.0 (66) | 5.4 | 0.1 (14) | 24.7 | 0.2 (2) | 44.5 |
| **Environmental stressors** | 7.9 (799) | 85.6 | 0.6 (38) | 98.3 | 2.7 (287) | 91.1 | 6.0 (76) | 89.6 |

NA= not applicable, SDQ = Strengths and difficulties questionnaire, KIVPA = short indicative questionnaire for psychosocial problems among adolescents, MHP = mental health problem

**Supplement figure 1 Calibration plots concerns for mental health problems (CMHP) (A, B, C) and extra healthcare use for CMHP (D, E, F)**

**A Population A B Population B C Population D**

**
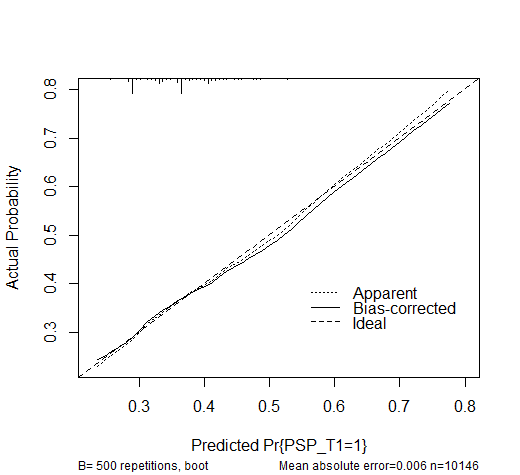

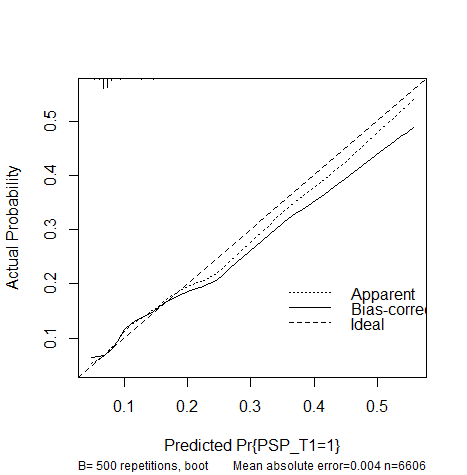

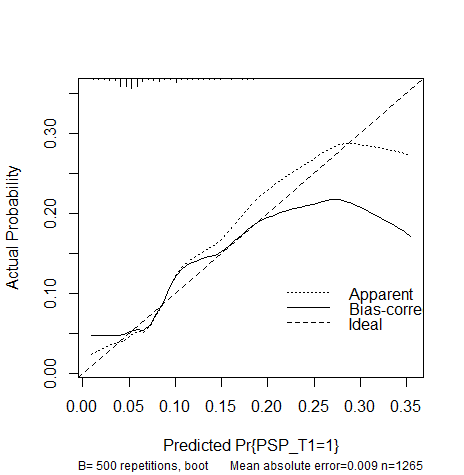

 D Population A E Population B F Population D

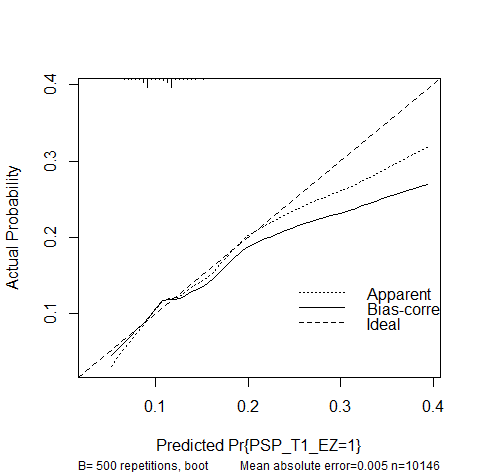

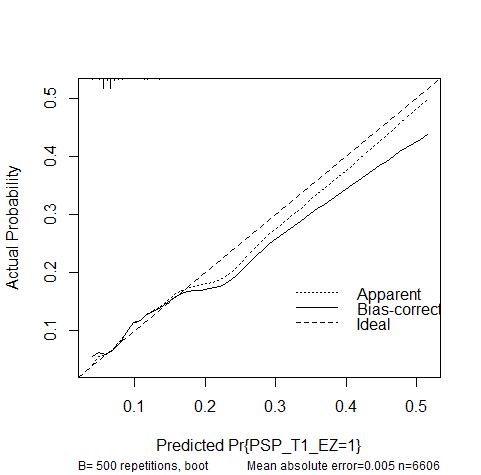

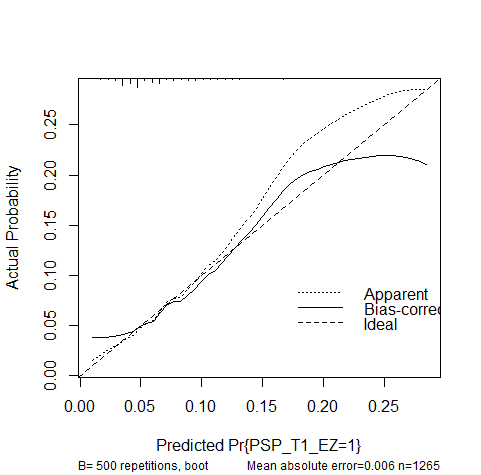
**

Calibration plots for predicting the 1-year risk of a first recorded CMHP (A, B, C) and extra healthcare use for CMHP (D, E, F). In each plot, the actual observation and predicted probabilities were drawn on the y- and x-axes respectively. The 45-degree dotted line depicts complete agreement between the actual and predicted probabilities.

Akkerman, S., Bakker, A.J.O., and Ontwikkeling (2012). Het leerpotentieel van grenzen. 25(1)**,** 15-19.

Cole, T.J., Bellizzi, M.C., Flegal, K.M., and Dietz, W.H. (2000). Establishing a standard definition for child overweight and obesity worldwide: international survey. *Bmj* 320(7244)**,** 1240.

De Nooijer, J., and De Vries, N.K.J.H.p.i. (2007). Monitoring health risk behavior of Dutch adolescents and the development of health promoting policies and activities: the E-MOVO project. 22(1)**,** 5-10.

Kist-van Holthe, J., Bulk-Bunschoten, A., Renders, C., L’Hoir, M., Kuijpers, T., and HiraSing, R. (2012). Richtlijn ‘Overgewicht’voor de jeugdgezondheidszorg.

Reijneveld, S.A., Vogels, A., Brugman, E., Van Ede, J., Verhulst, F.C., and Verloove‐Vanhorick, S.J.T.E.J.o.P.H. (2003). Early detection of psychosocial problems in adolescents: how useful is the Dutch short indicative questionnaire (KIVPA)? 13(2)**,** 152-159.

Sanders, M.R. (2008). Triple P-Positive Parenting Program as a public health approach to strengthening parenting. *Journal of family psychology* 22(4)**,** 506.

Schappin, R., De Graaf, I., and Reijneveld, S. (2017). Effectiviteit van Triple P in Nederland: stand van zaken en controverse. *Kind en adolescent* 38(2)**,** 75-90.

Statistics, U.I.f. (2012). *International standard classification of education: ISCED 2011.* UNESCO Institute for Statistics Montreal.

Van Widenfelt, B.M., Goedhart, A.W., Treffers, P.D., Goodman, R.J.E.c., and psychiatry, a. (2003). Dutch version of the Strengths and Difficulties Questionnaire (SDQ). 12(6)**,** 281-289.
